# On the Existence of Low-Mass Dark Matter and its Direct Detection

**DOI:** 10.1038/srep08058

**Published:** 2015-01-27

**Authors:** James Bateman, Ian McHardy, Alexander Merle, Tim R. Morris, Hendrik Ulbricht

**Affiliations:** 1 Quantum, Light and Matter, Physics and Astronomy, University of Southampton, Southampton SO17 1BJ, United Kingdom; 2 Astronomy, Physics and Astronomy, University of Southampton, Southampton SO17 1BJ, United Kingdom; 3 High Energy Physics Theory, Physics and Astronomy, University of Southampton, Southampton SO17 1BJ, United Kingdom; 4 Max-Planck-Institut für Physik (Werner-Heisenberg-Institut), Föhringer Ring 6, 80805 München, Germany

## Abstract

Dark Matter (DM) is an elusive form of matter which has been postulated to explain astronomical observations through its gravitational effects on stars and galaxies, gravitational lensing of light around these, and through its imprint on the Cosmic Microwave Background (CMB). This indirect evidence implies that DM accounts for as much as 84.5% of all matter in our Universe, yet it has so far evaded all attempts at direct detection, leaving such confirmation and the consequent discovery of its nature as one of the biggest challenges in modern physics. Here we present a novel form of low-mass DM *χ* that would have been missed by *all* experiments so far. While its large interaction strength might at first seem unlikely, neither constraints from particle physics nor cosmological/astronomical observations are sufficient to rule out this type of DM, and it motivates our proposal for direct detection by optomechanics technology which should soon be within reach, namely, through the precise position measurement of a levitated mesoscopic particle which will be perturbed by elastic collisions with *χ* particles. We show that a recently proposed nanoparticle matter-wave interferometer, originally conceived for tests of the quantum superposition principle, is sensitive to these collisions, too.

Dark Matter interacts at most weakly with ordinary matter. Most theories propose cross-sections for collisions of DM with nucleons which are typically very small, and therefore experimental attempts for its direct detection are usually performed with huge volumes containing many ordinary matter particles. Various different types of particles are discussed as candidates for DM. Very recent attempts to directly observe generic candidates such as supersymmetric DM^1^ or Kaluza–Klein DM^2^ did not reach conclusive results and it seems that DM still evades direct observation^3^. Also indirect experiments^4^ searching for annihilation products of DM or attempts to produce DM at the LHC at CERN^5^,^6^ have thus far not reported a clear signal, suggesting that WIMPs (Weakly Interacting Massive Particles)^7^, while being the natural guess for DM, might not exist in nature.

Alternative and typically very light DM candidates, such as axions^8^,^9^ or keV sterile neutrinos^10^,^11^, have been considered. Often, such particles decay very slowly or annihilate and so produce monoenergetic X-ray photons, which could be regarded as a smoking gun signature for such a type of DM. While dedicated satellite experiments have mostly derived strong limits, a recent detection of a line signal at 3.6 keV^12^,^13^ has attracted the attention of the community but would need to be solidified before a discovery was claimed. Further references on these matters are presented in the supplementary material.

This work is inspired by a recent suggestion that decoherence in matter-wave interferometry^14^,^15^ could be used as a sensitive detector for very light DM particles^16^. While much of the parameter space is excluded directly or indirectly by existing observations, we find a small range in which such a particle could exist and, with the properties so constrained, we make quantitative predictions for the expected decoherence. Such unorthodox suggestions are crucial to catalyse discussions between disparate areas of physics and facilitate progress in DM searches.

## Cosmological considerations

The decisive questions for a concrete DM candidate particle *χ* are whether it can be produced in the right amount in the early Universe and whether its velocity spectrum is not too warm to cause problems with cosmological structure formation. In Ref. 16, the concrete DM candidate was not specified, but putting the constraints from all sides together (see below and supplementary material for more details), we can narrow the possibilities down to a scalar particle *χ* with a mass of order *m_χ_* ≈ 100 eV and an elastic scattering cross-section on nuclei of *σ* ≈ 5 · 10^−29^ m^2^.

**Figure 1:**
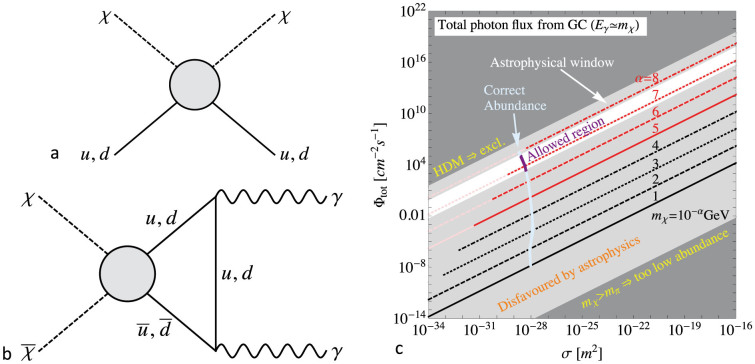
(a), Feynman diagram relevant for elastic scattering in a test particle. (b), Related diagram relevant for DM annihilation in the early Universe. (c), Integrated photon flux from the Galactic centre (if not shielded) versus collisional DM cross-section per nucleon, including all constraints which are applicable. The dark gray areas are excluded by consistency arguments, *i.e.*, the DM would either be hot for any choice of parameters (upper left triangle) or its mass would be too large for the suppression mechanism to apply to the annihilation diagram (lower right triangle). The light gray shaded regions are strongly constrained by astrophysical non-observations of the corresponding photons (see supplementary material), although some narrow line signals at particular energies may be difficult to fully exclude. The white patch is allowed by astrophysics. Each point on the red or black lines correspond to a certain DM abundance (see FIG. 6 in thesupplementary material), but the parts drawn in light colours would lead to hot DM scenarios, which are excluded as well. The region where the correct amount of DM is produced is marked by the light blue stripe, and the final resulting region allowed by all constraints is drawn in purple. This is what leads us to conclude that, putting all possible constraints together, the mass and scattering cross section of the *χ* particle should be around *m_χ_* ≈ 100 eV and *σ* ≈ 5 · 10^−29^ m^2^.

The standard process for DM production is *thermal freeze-out*, described further in the supplementary material. Cross-sections as needed for a detection in a matter-wave experiment^16^ would normally imply far too large annihilation, such that all such DM would be absent today. However, due to its small mass, the *χ* particle can only annihilate into *photons* at low temperatures, and this process is intimately connected to, but suppressed with respect to, the direct detection process, *cf.* FIGs. 1 a,b. Thus we can estimate the annihilation cross-section *σ*_ann_ of the DM particle in terms of the detection cross-section *σ* as 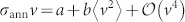, where a 
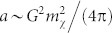
, *b* ~ *a*/24, and 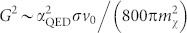, with *v* being measured in units of the speed of light *c*, *v*_0_ ~ 10^−3^, and 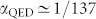. The additional loop-suppression of the annihilation keeps the DM abundance large enough to be consistent with observations.

## Particle Physics and Astrophysical constraints

The requirements for the *Z*^0^-boson and the neutral pion *π*^0^ not to decay into pairs 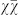 (and the Fermi pressure related Tremaine–Gunn bound^17^ for very light fermionic DM) force the particle to have vanishing spin (*i.e.* it is a scalar), and the requirement of DM not to be hot excludes very light DM masses, below 10 eV. The most obvious constraints come from missing energy signatures in colliders, in particular by the smoking gun signature of having a single photon in addition. (Strictly speaking, the energy may not necessarily be missing since our DM particle does interact considerably with nucleons. However, such a signal in a calorimetric experiment could easily be confused with other particles in jets.) Several detectors at LHC or previous experiments have reported strong bounds on such a signal^18^,^19^,^20^. However, using relatively general arguments about the ultraviolet completion behind the effective vertex displayed in FIGs. 1 a,b, one can see that these high energy bounds do not necessarily have to constrain the low energy vertex needed for DM, described further in the supplementary material. (The production of the *χ* particles is suppressed in the *s*-channel, or otherwise hidden in jets. Even though the interaction can in this sense be practically switched off at high energies, this does not affect early Universe cosmology since it does not matter whether the DM particle has been in equilibrium all along or has only entered equilibrium at some point before the freeze-out, as long as it is equilibrated for a sufficiently long time.)

The particle under consideration nevertheless has a comparatively large collisional cross-section *σ*, which means that it may be absorbed or reflected by the Earth's atmosphere. Furthermore, this could potentially lead to an additional mass contribution for celestial bodies (if the force between DM and ordinary matter is attractive) or to shifts in their trajectories (including precessions). Taking into account that the local DM energy density is tiny, only about 0.4 GeV/cm^3^, compared to the density of ordinary matter in a typical planet or star, the resulting mass shifts are tiny. For example, the Earth would collect about 1000 tonnes per year (which is a fractional increase of 10^−19^ in its mass per year). The *χ* DM pressure is of order *P* = 40 pPa. In our solar system, the Sun (and Jupiter) would be most affected by the resultant force, but it leads to the negligible acceleration ~ 4 · 10^−23^ ms^−2^. The order of magnitude (in radians) of precessional effects on the planets is given by the ratio of this DM force to the force from the Sun and, for example, for Earth this is an unmeasurably small *δθ* ~ 3 · 10^−17^ degrees/orbit. Finally, strong constraints arise from a potential annihilation signal arising from the same diagram as the production in the early Universe, *cf.* FIG. 1 b; however, the known observational bounds from several Earth- and space-based telescopes leave a window in which our DM candidate could still live. A detailed discussion can be found in the supplementary material. Thus, all astrophysical constraints are avoided naturally, and all constraints from particle physics do not apply (at least under relatively generic assumptions).

## Detection via elastic scattering

The presence of *χ* particles can be detected by the momentum they impart to a test particle via elastic collisions with the constituent nucleons; this recoil is measurable in either a classical detection scheme or via the reduction in fringe visibility in a matter-wave interferometer. Our candidate DM particles are sufficiently light and numerous that we do not expect to resolve individual scattering events; rather, we expect an overall drift in the direction of the DM, and a very small Brownian-like diffusion.

### Scaling with target particle size

**Figure 2:**
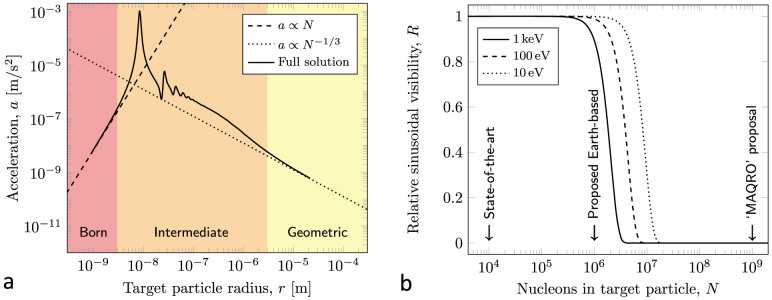
(a), Acceleration of a silicon test particle (nucleon number density 1.4 · 10^30^ m^−3^) across the size regimes for *χ* de Broglie wavelength 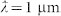. For small particles (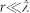), the Born approximation holds and acceleration is proportional to nucleon number; for large particles (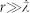), the force is proportional to projected area and thus increases slower than the inertia. In the intermediate regime (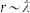), acceleration depends strongly upon the particle shape: for illustration we have chosen a spherical particle with an attractive interaction; the repulsive case is similar. Resonances, which distract from the main argument, have been smoothed by a few times their width. Similar plots are obtained for other de Broglie wavelengths, and the limiting cases are unaffected. (b), Reduction in sinusoidal fringe visibility due to elastic collisions for a range of *m_χ_*. Experiments with a similar geometry and path separation are indicated: state-of-the-art experiments have demonstrated 10^4^ nucleons^21^; an experiment with 10^6^ is proposed^22^; and space-based ‘MAQRO'^23^ will span the necessary range. For 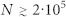, the Born approximation for scattering *χ* particles is not well satisfied and further theoretical work is needed to fully describe the decoherence.

A consequence of the low *χ* mass is that the de Broglie wavelength 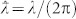 is large compared to the internuclear separation in normal matter, 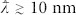. The *χ* hence scatters *coherently* from the constituent nuclei. For small particles under the Born approximation, all nuclei are subject to the same field from the incident *χ*, and we find an effective cross-section *σ*_eff_ = *σN*^2^. Conversely, for large particles, the flux is attenuated and the cross-section is the projected surface area *σ*_eff_ ∝ *N*^2/3^. In the intermediate regime, details of the interaction depend strongly on particle shape and on whether the underlying interaction is attractive or repulsive. For illustration, we consider a spherical particle with an attractive potential and we calculate the interaction via partial waves, described in further in the supplementary material; the expected acceleration *a* = *σ*_eff_*P*/*M*, where *M* is the particle mass, as shown in FIG. 2 a, reduces to the Born approximation and to the geometrical approximation in the respective limits. Details of size-dependent acceleration in the intermediate regime, if observed, will allow for an independent measurement of the *χ* DM pressure *P* and collisional cross-section *σ*.

### Dark Matter optics

For macroscopic objects, *χ* particles experience an average potential and, in close analogy with neutron optics^24^, the interaction may be described using a refractive index 
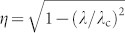
, where we identify the ‘critical wavelength' 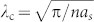, with *n* being the number-density of nucleons in the material, and the scattering length *a_s_* = ±2 fm is found via the low-energy limit in which 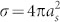. The uncertainty in sign (and thus whether *λ*_c_ is real or imaginary) arises because the cross-section is insensitive to whether the underlying interaction is attractive (−) or repulsive (+). For typical materials, 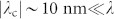, and we expect *χ* particles to be strongly reflected.

### Acceleration of a mesoscopic particle

**Figure 3:**
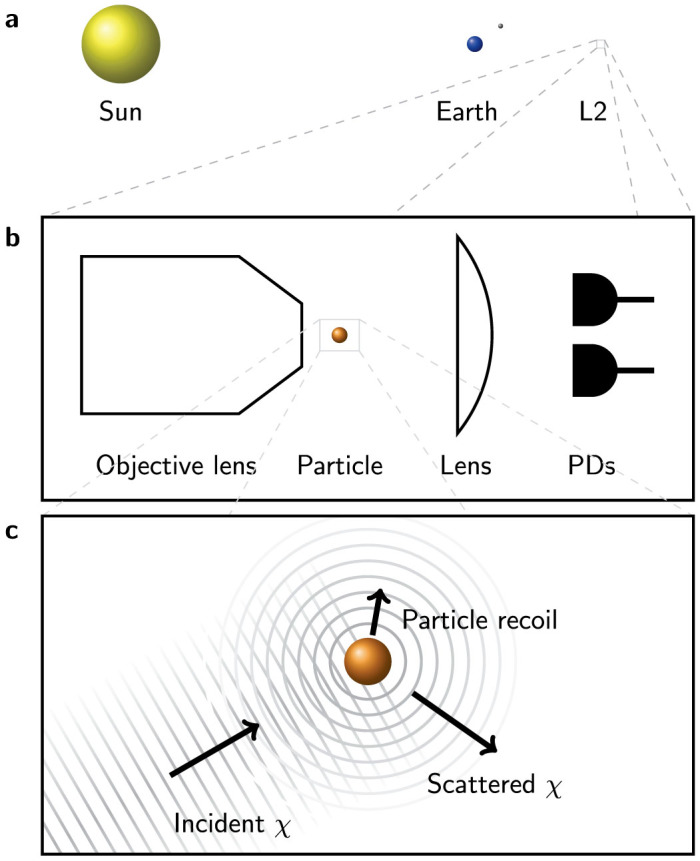
Illustration of the suggested experiment, the hardware for which can be provided by the proposed ‘MAQRO' space-craft^23^. (a), Location of the space-craft at Lagrange point 2 in the context of our solar system (not to scale). (b), Close-up of the optical arrangement: a compound objective lens provides high numerical aperture focusing for laser light to create a gradient-force dipole trap for a micron-scale particle. Light, which diverges strongly after the particle, is collected by a lens. Interference between the laser light and the light scattered coherently by the particle gives rise to a difference in intensity across the cross-section which, when measured by balanced photodiodes (PDs), provides sub-wavelength position information in three dimensions^25^. (c), A further close-up, showing *s*-wave scattering of a *χ* DM particle, with an approximately plane-wave incident wavefunction and an example scattering outgoing direction with the associated recoil of the test particle.

Given the possibility of a measurable effect upon nanometre-sized particles, and the uncertainty about whether *χ* particles will penetrate the Earth's atmosphere, we propose a space-based experiment, as illustrated in FIG. 3. Particle radii in the range 10 nm ≤ *r* ≤ 1 µm are expected to show accelerations 
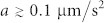
, with possibly much higher values and a rich size-dependent structure. Recently, 140 nm particles have been held in vacuum in a 120 kHz harmonic trap provided by a tight laser focus and feedback ‘cooled' to reduce the uncertainty in both their position (<1 nm) and velocity (500 µm/s)^25^. For a thermal state, the velocity uncertainty is the product of trap frequency and position uncertainty and, in ultra-high vacuum where gas collisions are negligible, one may decrease the trap frequency considerably; for a 10 kHz trap frequency, we expect a velocity uncertainty below 50 µm/s. After several minutes of free-flight under these conditions, the positional uncertainty will be sub-millimetre while acceleration from collisions with *χ* particles will give a millimetre-sized displacement. The effect is also observable without any such improvements; the displacement will be revealed in the statistics of position measurements.

### Matter-wave decoherence

The prediction of an acceleration is based upon the assumption that the Earth moves through the local DM distribution at some appreciable speed. However this local distribution is uncertain particularly for this yet-to-be-simulated DM candidate, so here we propose a detection scheme which does not rely on some overall drift.

Elastic scattering events can be interpreted as revealing partial which-way information or, in a more complete treatment including recoil, diffusing momentum in a quantum Brownian Motion^26^. While individual collisions may not affect the visibility significantly, many such events will have a measurable effect. A proposed space-based matter-wave nanoparticle interferometer^23^ will be sensitive to this decoherence mechanism and the effect can be controllably extinguished by shielding the nanoparticle from the DM flux. We analyse the decoherence for a similar interferometer^22^, where a nanoparticle, prepared in a thermal state of a harmonic oscillator via feedback cooling, provides a point-like source for a near-field (Fresnel region) Talbot interferometer using a phase grating of period Λ provided by a standing light-wave.

The overall fringe pattern is found via a Wigner function phase-space treatment and is expressed as a Fourier series, the first order of which may be robustly extracted from experimental data by fitting to a sinusoid. Each decoherence mechanism reduces this amplitude by a factor 
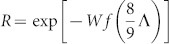
, where *W* = *cv*_0_*σ*_eff_ *τ ρ_χ_*/*m_χ_* is the total number of events (the flux multiplied by the cross-section and the duration *τ* of the experiment), the numerical factor ^8^/_9_ comes from the geometry of the experiment, and *f*(*x*) describes the spatial resolution of each event, as described in the supplementary material. For 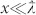, individual events affect the state little and multiple events are necessary to cause a measurable decoherence; for 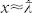, each event reduces visibility by approximately one half. A silicon particle of internal temperature below 50 K in ultra-high vacuum has negligible decoherence from the two important mechanisms, black-body radiation and gas collisions, and the reduction in visibility is dominated by decoherence from collisions with *χ* particles; this is shown in FIG. 2 b and we see that this decoherence is significant for experimentally accessible masses.

## Concluding remarks

We predict a light form of DM and we have argued that it is possible that this specific form of DM, the particle *χ*, would not have been observed in any experiment so far. We identify the mass range of *χ* and its collisional cross-section. We hope that this will catalyse further developments of more detailed particle theories and allow for more precise predictions of the properties. While both of the optomechanical experiments which we have proposed are space-based, the possibility of Earth-based detection is the topic of ongoing research; the prospects for such detection depend on the details of the particle theory, which is yet to be developed, and on the details of *χ* particle interaction with the atmosphere. Both the modulation of the DM flux as expected on Earth due to planetary motion and the possibility of extinguishing the flux with a mechanical shutter provide clear experimental signatures for identification of *χ*. Observation of a size-dependent acceleration would reveal far more details about the nature of these particles.

Experimentally, the possibility of defining a refractive index for the interaction of *χ* with ordinary matter allows for the implementation of optical elements to manipulate, guide, and even suppress reflections of DM beams. We can hope to greatly increase the local density of this type of DM, if it exists. Furthermore, complementary detection techniques should be studied as well as the possibility for direct *χ* production at low energy, high intensity photon colliders^27^.

In this letter, we have discussed many aspects of the new DM candidate *χ* and tried to be as complete as possible, but some points lie beyond the scope of this work. For example, one would in principle need to study *all* possible bounds arising from the many known mesons, but even the bound from pion decay would not be too strong (if it applied) and anything beyond that is less clean and/or would rely on additional assumptions, such as the coupling of *χ* to *s*-quarks. Furthermore, the presence of the *χ*'s in the early Universe could modify Big Bang Nucleosynthesis (BBN), but it is hard to estimate the effect without a detailed simulation, because not only would the *χ*'s comprise additional radiation but they might also modify the formation of nuclei due to the coupling to *u*- and *d*-quarks. Thus, depending on the details, the effect of including *χ* could be anywhere between destroying the success of BBN and maybe even solving the lithium problem. Detailed simulations of the dynamics of global cosmic structures or of objects such as the bullet cluster are beyond the immediate scope of this work. All these aspects could yield additional bounds and/or benefits for our setting, and they hence offer multiple starting points for future research projects scrutinising our proposal.

## Author Contributions

A.M., T.R.M. and H.U. conceived the possibility of this D.M. candidate. A.M. discovered the suppression mechanism. A.M. and T.R.M. analysed the D.M. production and worked out the constraints from the particle physics side. I.McH. provided the constraints from X-ray astronomy. T.R.M., J.B. and H.U. conceived the detection. J.B. calculated acceleration and decoherence, and J.B. and H.U. designed the experiment. All authors discussed the results and wrote the manuscript.

## Supplementary Material

Supplementary Information Supplementary Information
